# Genetic diversity in the partial sequence of the HIV-1 *gag* gene among people living with multidrug-resistant HIV-1 infection

**DOI:** 10.1590/S1678-9946202466035

**Published:** 2024-06-07

**Authors:** Cecília Salete Alencar, Ester Cerdeira Sabino, Ricardo Sobhie Diaz, Alfredo Mendrone-Junior, Anna Shoko Nishiya

**Affiliations:** 1 Universidade de São Paulo, Faculdade de Medicina, Hospital das Clínicas, Laboratório de Medicina Laboratorial (LIM-03), São Paulo, São Paulo, Brazil; 2 Universidade de São Paulo, Faculdade de Medicina, Instituto de Medicina Tropical de São Paulo, São Paulo, São Paulo, Brazil; 3 Universidade de São Paulo, Faculdade de Medicina, São Paulo, São Paulo, Brazil; 4 Universidade Federal de São Paulo, Laboratório de Retrovirologia, São Paulo, São Paulo, Brazil; 5 Fundação Pró-Sangue Hemocentro de São Paulo, São Paulo, São Paulo, Brazil; 6 Universidade de São Paulo, Faculdade de Medicina, Hospital das Clínicas, Departamento de Hematologia, Laboratório de Investigação Médica em Patogênese e Terapia Dirigida em Onco-Imuno-Hematologia (LIM-31), São Paulo, São Paulo, Brazil

**Keywords:** Gag HIV-1, Subtypes, Recombination, Resistance mutations, Protease inhibitor, Virological failure

## Abstract

The group-specific antigen (*gag*) plays a crucial role in the assembly, release, and maturation of HIV. This study aimed to analyze the partial sequence of the HIV *gag* gene to classify HIV subtypes, identify recombination sites, and detect protease inhibitor (PI) resistance-associated mutations (RAMs). The cohort included 100 people living with HIV (PLH) who had experienced antiretroviral treatment failure with reverse transcriptase/protease inhibitors. Proviral HIV-DNA was successfully sequenced in 96 out of 100 samples for *gag* regions, specifically matrix (p17) and capsid (p24). Moreover, from these 96 sequences, 82 (85.42%) were classified as subtype B, six (6.25%) as subtype F1, one (1.04%) as subtype C, and seven (7.29%) exhibited a mosaic pattern between subtypes B and F1 (B/F1), with breakpoints at p24 protein. Insertions and deletions of amino acid at p17 were observed in 51 samples (53.13%). The prevalence of PI RAM in the partial *gag* gene was observed in 78 out of 96 PLH (81.25%). Among these cases, the most common mutations were R76K (53.13%), Y79F (31.25%), and H219Q (14.58%) at non-cleavage sites, as well as V128I (10.42%) and Y132F (11.46%) at cleavage sites. While B/F1 recombination was identified in the p24, the p17 coding region showed higher diversity, where insertions, deletions, and PI RAM, were observed at high prevalence. In PLH with virological failure, the analysis of the partial *gag* gene could contribute to more accurate predictions in genotypic resistance to PIs. This can aid guide more effective HIV treatment strategies.

## INTRODUCTION

The genetic diversity and evolution of HIV-1 pose a significant challenge to the development of vaccines and diagnostic assays, as well as impacting drug resistance and responses to antiretroviral treatment^1-4^. The HIV-1 group M (main) has diversified into distinct subtypes labelled A, B, C, D, F, G, H, J, and K, also including recombinants between subtypes, known as circulating recombinant forms (CRFs) and unique recombinant forms (URFs)^5^. Globally, subtype C constitutes 46.6% of all HIV-1 infections, with subtype B at 12.1%, subtype A at 10.3%, CRF02_AG at 7.7%, CRF01_AE at 5.3%, subtype G at 4.6%, and subtype D at 2.7%^6^. The CRF classification is established by analyzing complete HIV genome sequences or genes responsible for encoding the group-specific antigen (*gag*), envelope, and polymerase (pol)^6^.

The HIV *gag* protein plays a key role in virus particles’ assembly, release, and maturation^7^. Successful maturation requires the systematic proteolytic cleavage of the *gag* polyprotein by the viral protease. This process is necessary for releasing the matrix (p17), capsid (p24), nucleocapsid (NC), and p6 proteins^8^. Protease inhibitors (PIs) are potent antiretroviral drugs designed as structural analogues of *gag* cleavage sites, aiming to hinder the viral maturation process^9,10^. However, resistance to protease inhibitors may develop in cases of treatment failure, particularly when using unboosted PIs or in the context of transmitted drug resistance. Consequently, mutations in both the protease and *gag* genes can contribute to resistance. Notably, resistance may also arise due to mutations in the *gag* substrate alone, independent of the protease enzyme^11,12^.

This study aimed to analyze the partial sequence of the HIV *gag* gene to verify genetic diversity, identify recombination breakpoints, and assess protease inhibitor (PI) resistance-associated mutations (RAM) among people living with HIV (PLH) who have already failed to reverse transcriptase/protease inhibitors treatment.

## MATERIALS AND METHODS

### Study population

This study included samples from a previous study that sequenced part of the envelope gene to verify subtypes and transmitted drug resistance to HIV entry inhibitors among PLH failing to reverse transcriptase (RT)/protease inhibitors (PI) treatment^13^. These samples already had the DNA extracted from 100 consecutive PLH from a public outpatient clinic (Centro de Referencia e Treinamento DST/AIDS de Sao Paulo) in the Sao Paulo city (latitude −23.5489, longitude −46.6388) after approval by the institution's research ethics committee. Inclusion criteria were viral load ≥ 5,000 copies/ml, PLH older than 18 years failing treatment with nucleoside and non-nucleoside reverse transcriptase inhibitors (NRTI and NNRTI) and protease inhibitors (PI), and no previous exposure to others antiretroviral classes. The subjects were invited to participate during blood collection for a pol genotype test routinely performed for PLH failing antiretroviral treatment. After signing an informed consent form, participants were interviewed about clinical and demographic characteristics, and an extra 10 ml of blood was collected. Medical charts were also reviewed to confirm previous antiretroviral exposure.

### DNA extraction and amplification

The DNA was obtained from 200 µl of blood samples using a QIAamp DNA Blood Mini Kit (QIAGEN, GmbH, Hilden, Germany) following the manufacturer's instructions. A fragment with approximately 1,052 bp containing the *gag* matrix (p17) and capsid (p24) region was amplified. The primers Gag-F1 (outer) 5’-TTTGACTAGCGGAGGCTA-3’ and Gag-R1 (outer) 5’-ACTCCCTGACATGCTGTC-3’ were used in the first round amplifications, and the primers Gag-F2 (inner) 5’-AGATGGGTGCGAGAGCGT-3’ and Gag-R2 (inner) 5’-ACATGCTGTCATCATTTCTTC-3’^14^ were used in the second round amplifications. The PCR mixture was performed using 0.4 mM dNTPs (Amersham Pharmacia Biotec, Piscataway, NJ), 2.5 mM MgCL2 (Invitrogen, Carslbad, CA), 0.2 pmol/µl of each primer, and 1.5 U of Taq-DNA polymerase (Invitrogen) for the final volume of 50 µl. Amplification for the first round was one cycle of 94 °C for 1 min., followed by 35 cycles of 94 °C for 45 s, 55 °C for 45 s, and 72 °C for 2 min, with a final extension of 72 °C for 10 min. The second round of amplification included an initial cycle of 94 °C for 1 min, followed by 35 cycles of 94 °C for 1 min, 55 °C for 1 min, and 72 °C for 1:30 min, and a final extension of 72 °C for 10 min.

### HIV-1 subtype, mutations, and recombination analyses

The PCR product of the second amplification was purified using a QIAquick Kit (QIAGEN) following the manufacturer's instructions, and directly sequenced using a Big Dye Terminator Cycle Sequencing Ready Reaction Kit (Applied Biosystems, Foster City, CA) according to manufacturer procedure. The primers Gag-F2 5’-AGATGGGTGCGAGAGCGT-3’ and Gag_R-2 5’-ACATGCTGTCATCATTTCTTC-3’^14^ were used, and the sequences were edited using the Sequencher software program (version 4.1.4, Gene Codes Corporation, Ann Arbor, MI, USA). The sequences were deposited in the GenBank under access numbers KF715272 and KF715367.

HIV-1 subtype and mutations analyses were performed using the Rega HIV Subtyping Tool Version 3.46 (https://www.genomedetective.com/app/typingtool/hiv/)^15^. The *gag* cleavage site mutations associated with PI resistance were: V128I/T/A and Y132F (in matrix), and in non-cleavage site were: E12K, V35I, E40K, G62R, L75R, R76K, Y79F, T81A K112E, and G123E (in matrix) and M200I and H219Q/P (in capsid)^11,16^. The HIV subtype recombination breakpoints were detected using the jumping profile hidden Markov model (jpHMM)^17^.

## RESULTS

### Clinical and demographic characteristics of PLH

The median age of PLH was 45 years (range 21–65), most (82%) were men, 42% had complete secondary education, and 56% were retirees. Heterosexual exposure was the most prevalent (49%), followed by homosexual men (40%) and bisexual men (11%). The median duration of HIV-1 infection was 11 years (range 5–21), with 51% being asymptomatic.

Medical records were reviewed and exposure to antiretrovirals was verified. HIV pol (RT and P genes) genotypic resistance test presented a total of 55% of resistance to the three classes together NNRTI/NRTI/PI; 24% only to PI, NNRTI/PI, or NRTI/PI; and 21% to NNRTI and/or NRTI. Thus, the prevalence of PLH with HIV PI RAM in the protease gene was 79% (55% plus 24%).

The viral load exceeded 1,000,000 copies/mL of plasma in 36% of subjects. The median number of sexual partners was eight individuals per year. Intravenous drug use was reported by 9%, and unprotected intercourse was reported by 84%, with 60% of those reporting unprotected intercourse with sex workers.

Primary associated diseases included pneumonia (21%), tuberculosis (18%), toxoplasmosis (5%), and sexually transmitted diseases such as herpes (18%), viral hepatitis B and C (22%), syphilis (13%), and HPV (12%).

### Molecular characteristics of HIV

HIV subtypes were identified by analyzing the partial *gag* region, specifically the matrix (p17) and capsid (p24). Sequencing was successful in 96 out of 100 samples, achieving a 96% success rate. Of these, 82 (85.42%) were classified as subtype B, six (6.25%) as subtype F1, one (1.04%) as subtype C, and seven (7.29%) as mosaics, consisting of a combination of B and F1.

**Figure 1:**
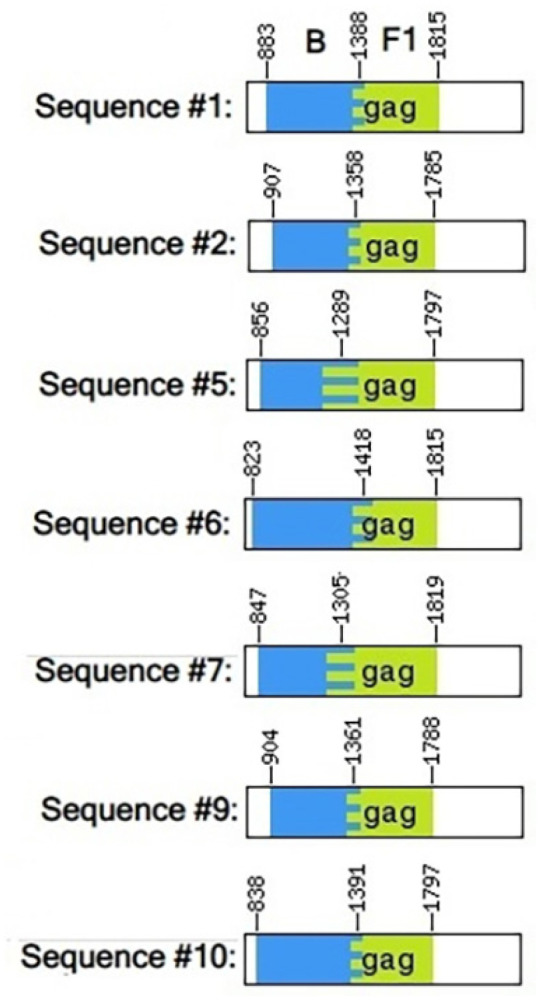
The BF1 recombination occurred within the region that encodes the capsid p24 protein, spanning amino acid positions 150 to 180 (or nucleotide positions with reference to the HXB2: 1289, 1305, 1358, 1361, 1388, 1391, and 1418).

Recombination events were observed exclusively in the region encoding the capsid (p24) protein, specifically at positions spanning amino acids 150 to 180 (or nucleotide positions based on the HXB2 reference: 1289, 1305, 1358, 1361, 1388, 1391, and 1418) (Figure 1).

In the protein-coding region of the matrix (p17), 53.13% (51/96) of the samples exhibited deletions and insertions. Within this subset, 46 (47.92%) presented insertions, 4 (4.17%) exhibited deletions, and one (1.04%) showed simultaneous insertions and deletions. Subtype distribution indicated that insertions were predominantly found in subtype B samples (43), followed by two recombinants and one subtype F. Deletions were identified in three subtype F samples and one subtype B sample. A subtype C strain displayed concurrent deletions and insertions. Insertions occurred at amino acid positions 113 to 130, and deletions were observed at amino acid positions 122 to 127, based on the HIV HXB2 reference in the p17 protein.

**Figure 2:**
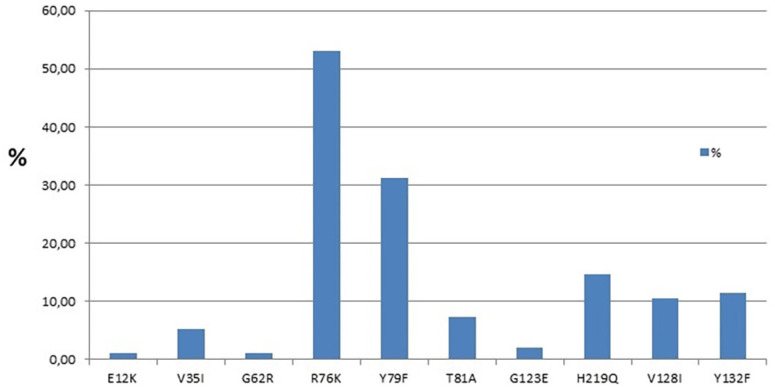
Frequency of drug resistance-associated mutations in the HIV *gag* gene at non-cleavage sites (E12K, V35I, G62R, R76K, Y79F, T81A, G123E, and H219Q) and cleavage sites (V128I and Y132F).

The prevalence of PI RAM in the partial *gag* gene was 78/96 (81.25%) in the HIV of PLH who experienced virologic treatment failure with NRTI and NNRTI and PI. Among these, 59/96 (61.45%) exhibited mutations in non-cleavage sites, and 19/96 (19.79%) had mutations in both cleavage and non-cleavage sites concurrently. Cleavage site mutations identified in the *gag* gene included V128I (10.42%) and Y132F (11.46%) in the matrix. Non-cleavage site mutations comprised E12K (1.04%), V35I (5.21%), G62R (1.04%), R76K (53.13%), Y79F (31.25%), T81A (7.29%), and G123E (2.08%) in the matrix, and H219Q (14.58%) in the capsid (Figure 2).

## DISCUSSION

In this study, we analyzed the partial *gag* genome, region of matrix (p17) and capsid (p24) among PLH experiencing treatment failure with NRTI, NNRTI, and PI at an outpatient clinic in the Sao Paulo city. Our findings revealed a predominance of subtype B, followed by BF mosaic, F1, and C. In the Southeast region of Brazil— where Sao Paulo city is located, the predominant HIV subtypes have been found to be B, followed by BF and F1^18^, which corroborate our findings.

We observed a 7-fold higher prevalence of BF recombinants in *gag* (7%) than in the previously studied envelope gene (1%)^13^. Consistent with our findings, Souza *et al.* ^19^ also reported preferential recombination sites within the HIV genome, primarily in the polymerase, followed by *gag*, and finally in the envelope gene. That study also described that the highest frequency of recombination breakpoints was observed in the capsid p24^19^, confirming our findings.

More than 80% of PLH who experienced treatment failure presented PI resistance-associated mutations in the *gag* gene. The most frequent mutations observed were R76K, Y79F, and H219Q at the non-cleavage sites and V128I and Y132F at cleavage sites. Mutations at cleavage sites can directly affect the interaction with PI, whereas mutations at non-cleaved sites can contribute to restoring viral fitness, thus improving the access of the viral protease to the cleavage site^11,20^. Comparatively, a study conducted in Mexico showed a higher prevalence of the R76K and H219Q mutations and lower prevalence of the V128I and Y132F mutations among treatment-naïve individuals^21^.

In addition to the mutations associated with resistance to PIs, more than half of the samples presented deletions and insertions in the protein-coding region of the matrix (p17), presenting greater sequence variation compared to the capsid. This pattern is consistent with similar studies that have also reported more quasispecies diversity, polymorphisms, deletions, and insertions in the matrix p17^21,22^.

Another study used the inverse substrate-protease analysis to determine *gag* mutations associated with PI resistance upon reversion to wild type after analytical antiretroviral treatment interruption among PLH experiencing failing PI treatment^23^. In this study, the Y132F mutations at the *gag* cleavage site were identified. However, most of these mutations did not revert to the wild type after 12 months of analytical treatment interruption (ATI). Conversely, mutations outside the *gag* cleavage sites, specifically R76K and Y79F, exhibited a reversion to wild type in most cases after ATI^23^.

This study used samples collected in 2006 from a previous study. We performed PCR and sequencing of part of the *gag* gene using the Sanger methodology and identified the subtypes and recombinants at that time, which were stored for years. Researchers have linked mutations in *gag* and resistance to protease inhibitors; in this study, we resumed the research. Although we did not use next-generation sequencing, more recent sampling, and a larger region of the *gag* gene, our study found some interesting results. Other limitation of the study was the absence of an analysis of the NC and p6 regions of the *gag* gene. This is noteworthy since mutations in NC/p1 and p1/p6 cleavage sites are also associated with PI resistance^24^. However, it becomes evident that characterizing the gag profile among PLH failing PIs would enhance the positive predictive value of genotypic resistance testing results aimed to determine the decrease of susceptibility of HIV to PIs. These results still need to be validated with phenotypic resistance testing and results of clinical trials. However, since 79% of our samples presented PI RAM in the protease region and the prevalence of PI RAM found in the *gag* gene was 81%, we suggest that inspecting gag mutations may be more accurate than inspecting protease to infer about PI resistance in this specific population. Additionally, as alternative examples, we noted that mutations in the RNAse gene are associated with resistance to NRTIs and NNRTIs^25^. Moreover, mutations at the 3’ polypurine tract region of the Nef gene lead to resistance to integrase strand transfer inhibitors^26^.

The current PI prevalence in PLH is very low but remains important in some situations. In the second-generation integrase strand transfer inhibitor, PIs are used as alternative regimens or in salvage therapy regimens. In this context, boosted PI was found to prevent selection of PI resistant strains. However, PI resistance is still important since there are PLH that have been exposed to unboosted PIs in the beginning of the HIV epidemic, and few PLH harbor PI transmitted drug resistance HIV strains^27^. Sensing the importance of PI resistance and the importance in generating science knowledge involving the relationship between the PI RAMs and *gag* cleavage site mutations, we decided to pursue a historical set of samples that had been already characterized for PI RAMs from when PI RAM was much more prevalent^13^. Thus, in this study, by investigating variations in the HIV *gag* gene among PLH experiencing virological failure after treatment with reverse transcriptase and PIs, we found a high genetic diversity, with numerous recombinant breakpoints, insertions, and deletions. Notably, identifying PI resistance mutations in the *gag* gene could significantly improve the accuracy of predicting positive outcomes in genotypic PI resistance tests for individuals experiencing failure in antiretroviral therapy. This research underscores the significance of comprehending shifts in genetic diversity and the emergence of drug-resistant strains pathways, allowing the future inclusion of *gag* genetic profile in resistance analysis algorithm for more accurate interpretations of the genotypic corelates of antiretroviral as a tool for clinical assistance.
